# Design and Manufacture of a Low-Cost Microfluidic System for the Synthesis of Giant Liposomes for the Encapsulation of Yeast Homologues: Applications in the Screening of Membrane-Active Peptide Libraries

**DOI:** 10.3390/mi12111377

**Published:** 2021-11-10

**Authors:** Saúl C. Gómez, Valentina Quezada, Isabella Quiroz, Carolina Muñoz-Camargo, Johann F. Osma, Luis H. Reyes, Juan C. Cruz

**Affiliations:** 1 Department of Biomedical Engineering, Universidad de los Andes, Cra. 1E No. 19a-40, Bogotá 111711, Colombia; sc.gomez11@uniandes.edu.co (S.C.G.); v.quezada@uniandes.edu.co (V.Q.); i.quiroz@uniandes.edu.co (I.Q.); c.munoz2016@uniandes.edu.co (C.M.-C.); 2 Department of Electrical and Electronic Engineering, Universidad de los Andes, Cra. 1E No. 19a-40, Bogotá 111711, Colombia; 3 Department of Food and Chemical Engineering, Universidad de los Andes, Cra. 1E No. 19a-40, Bogotá 111711, Colombia

**Keywords:** Giant Unilamellar Vesicles, micromixers, multiphysics simulation, chitosan microparticles

## Abstract

The discovery of new membrane-active peptides (MAPs) is an area of considerable interest in modern biotechnology considering their ample applicability in several fields ranging from the development of novel delivery vehicles (via cell-penetrating peptides) to responding to the latent threat of antibiotic resistance (via antimicrobial peptides). Different strategies have been devised for such discovery process, however, most of them involve costly, tedious, and low-efficiency methods. We have recently proposed an alternative route based on constructing a non-rationally designed library recombinantly expressed on the yeasts’ surfaces. However, a major challenge is to conduct a robust and high-throughput screening of possible candidates with membrane activity. Here, we addressed this issue by putting forward low-cost microfluidic platforms for both the synthesis of Giant Unilamellar Vesicles (GUVs) as mimicking entities of cell membranes and for providing intimate contact between GUVs and homologues of yeasts expressing MAPs. The homologues were chitosan microparticles functionalized with the membrane translocating peptide Buforin II, while intimate contact was through passive micromixers with different channel geometries. Both microfluidic platforms were evaluated both in silico (via Multiphysics simulations) and in vitro with a high agreement between the two approaches. Large and stable GUVs (5–100 µm) were synthesized effectively, and the mixing processes were comprehensively studied leading to finding the best operating parameters. A serpentine micromixer equipped with circular features showed the highest average encapsulation efficiencies, which was explained by the unique mixing patterns achieved within the device. The microfluidic devices developed here demonstrate high potential as platforms for the discovery of novel MAPs as well as for other applications in the biomedical field such as the encapsulation and controlled delivery of bioactive compounds.

## 1. Introduction

Biological membranes are a selective natural barrier that regulates the entry of therapeutic agents due to their amphipathicity, which renders them impermeability for most peptides, proteins, and oligonucleotides while preserving the intracellular contents [1,2]. This has motivated the search for new molecules capable of penetrating the membrane for the transport and release of both hydrophilic and hydrophobic molecules intracellularly. Some of these penetrating molecules include different types of proteins and peptides [2]. Besides, penetration, some peptides interact with cell membranes strongly, leading to substantial disorganization (and generally exhibiting antimicrobial activity), intercalation, or even fusion. All these peptide sequences have been group into a big family called membrane-active peptides (MAPs) [3]. Typically, MAPs are sequences of 4–40 positively charged amino acids at a neutral pH [4]. There are two major classes of MAPs; antimicrobial peptides (AMPs), which kill cells, and cell-penetrating peptides (CPPs), which can carry cargoes through lipid bilayers [3].

AMPs have been considered as an alternative to antibiotics for the treatment of bacterial infections either alone or in conjunction with conventional antibiotic-based therapies [1,5,6,7]. This has been attributed to AMP’s wide spectrum of activity against several pathogens. Antibiotics generally act on essential survival processes of bacteria, such as inhibition of cell wall synthesis, interference with the synthesis of essential proteins, and disruption of membrane integrity [8]. However, bacteria have developed defense mechanisms through natural processes that promote resistance against them. According to the World Health Organization (WHO), the rapid spread of drug resistance throughout the world is leading us to a post-antibiotic age, where contracting infections will be more frequent and their mortality will increase almost exponentially, with an estimated ten million deaths per year by 2050 [9]. The resistance processes occur when the antibiotic molecule loses its ability to effectively inhibit bacterial growth even under therapeutic concentration levels [10,11]. This mechanism has been associated with horizontal gene transfer where different species of bacteria acquire resistance relatively easily [9,11]. This further complicates this major global health care problem and emphasizes the need for complementary solutions that go even beyond awareness campaigns on the correct use of antibiotics.

CPPs and AMPs are sequences between 5–50 amino acids. CPPs are generally positively charged, facilitating the generation of electrostatic interactions with cell surface components that are negatively charged, producing membrane translocation [1,4,12,13,14]. Conversely, it has been demonstrated that CPPs are a potential tool for the delivery of bioactive molecules such as plasmids, oligonucleotides, peptidic nucleic acid (PNA), proteins, fluorescent agents, drugs, and even other peptides, making them suitable for antimicrobial, antifungal and antiparasitic applications [1,3]. Similarly, AMPs are rich in hydrophobic residues, with an amphipathic structure that exhibits a broad spectrum of activity against microorganisms [15,16,17,18,19,20]. AMPs interact with the negatively charged components of the cell membrane, modulating immune response and controlling shrinkage, without compromising other eukaryotic membranes [17,19,21]. AMPs act on the lipid bilayer as a detergent that solubilizes the components of the membrane, turning them into micelles and forming pores that allow them to reach the intracellular space. Consequently, transient permeabilization of the membrane is achieved and a cytoplasmic leakage initiates a cell death process [18,20,22,23]. In addition, it has been found that AMPs can interfere with vital processes intracellularly, inhibiting proteases and the processes of cell division and biosynthesis of proteins, nucleic acids, and cell wall components [24]. These interactions with various cellular components of the bacteria make them potential candidates to replace antibiotics, with the advantage that they are less prone to bacteria developing resistance to them [25].

Different studies have demonstrated the ability of CPPs to disrupt bacterial membranes, due to the presence of arginine residues, which can interact strongly with membranes destabilizing them [12,26,27]. Also, some AMPs have the ability to translocate into the cytoplasm without damaging the membrane, so they can be used as antibiotics and as precursors of drug transporters [2,28,29]. This is mainly because CPPs and AMPs share certain features such as secondary structure, size, charge, and, in general, their composition, which explains their strong interaction with negatively charged membranes [3,12]. This means that, in theory, all cationic CPPs are AMPs and vice versa, where, sometimes, only one mutation is sufficient to modify their membrane activity [3]. This important versatility makes MAPs a promising alternative to address problems of great relevance in the biomedical field, such as the development of systems for the controlled release of drugs and antimicrobial agents to combat antibiotic resistance. However, the isolation of natural peptides is a complex, inefficient and costly task in terms of time and economic resources [16,17]. This lack of simple and biologically relevant methods for comprehensively screening peptide libraries makes their synthesis on a large scale difficult compared to antibiotics [30].

This issue has motivated the development of different high-throughput screening techniques (HTS) that allow the identification of pharmacologically or biologically active compounds in a large-scale process, facilitating the parallel analysis of millions of reactions in relatively short periods [31]. However, these require a large number of samples and reagents, as well as sophisticated detection schemes that considerably increase the costs associated with their manufacture [32,33]. As a solution to this, microfluidic systems have been extensively studied for the screening of bioactive compounds, such as DNA, proteins, enzymes, receptors, and AMPs, demonstrating in all cases great advantages, mainly due to the ability to perform thousands of reactions on the scale from nanoliters to femtoliters, replacing automation mechanisms, using small volumes of samples, reducing experimentation costs and increasing the overall performance of the screening method [34,35,36,37]. Currently, different techniques have been implemented from the field of microfluidics for the screening of MAPs, among which three main strategies stand out: droplet-based approaches, combinatorial microarrays, and membrane-based approaches [38]. However, it is important to highlight that the field of AMPs has received special attention in recent years due to the need to develop new antimicrobials, where a delay in the publication of new studies using these techniques for the screening of CPPs is evident [3].

The droplet-based technique consists of compartmentalization of reagents in picoliter volume emulsion droplets integrated with detection mechanisms such as fluorescence-activated cell sorting (FACS) that can be implemented for MAPs screening applications [39,40,41,42]. In the case of AMPs, these are encapsulated in droplets together with a set of reporter cells and reagents, which, when binding with the specific ligand, emit fluorescence that is used for the classification and purification of these candidates [38]. This approach has proven useful for several applications including, the unicellular analysis of peptide uptake in cancer cells [43], the high-throughput identification of peptides agonists of G protein receptors [44], the screening of libraries generated in the droplets through the interaction with microbial cells for evaluating antimicrobial activity [39], and the screening of compounds secreted by yeasts such as *Yarrowia lipolytica* [45,46]. Regarding CPPs, arrays of droplets have been immobilized into microfluidic systems to trap peptides flowing through the microchannels [43]. Additionally, combinatorial chemistry techniques have been implemented to study biomolecular interactions of libraries of mixtures of immobilized proteins or peptides with several chemical compounds prepared on a large scale in a single experiment [47]. This method has been successful in the screening of non-rational combinatorial libraries of peptides [48,49], peptide libraries based on magnetic separation of biotinylated aminopeptidases (APN) [50], and impact printing microfluidic platforms for the combinatorial synthesis of chemical compounds [51].

Finally, the membrane-based approach has mainly exploited two platforms, namely, artificial planar lipid bilayers and liposomes [52]. Artificial planar lipid bilayers have been used to determine the interaction of AMPs with lipid bilayers and particularly for studying the mechanisms of pore-forming proteins and peptides [53,54]. Also, giant liposomes known as Giant Unilamellar Vesicles (GUVs) synthesized via microfluidics-based methods such as Octanol-assisted Liposome Assembly (OLA) [55] have demonstrated versatility for the screening of AMPs, CPPs, and translocating drug molecules [52,56,57,58]. Thus, considering the potential of GUVs and their cell-like characteristics, it is possible to consider them for studying the translocation activity of CPPs by looking at their direct interaction within a system providing a sufficient mixing level. An interesting option is the passive micromixers where interaction is promoted within carefully designed microchannels in terms of geometry, mixing time (MT), mean residence time (MRT), and trajectory length (TL). The MT is defined as the time required to reach a steady mixture, the MRT corresponds to the mean of the time distribution spent by a particle within the system, and the TL is the mean of the length distribution for a particle traveling within the geometry.

**Figure 1:**
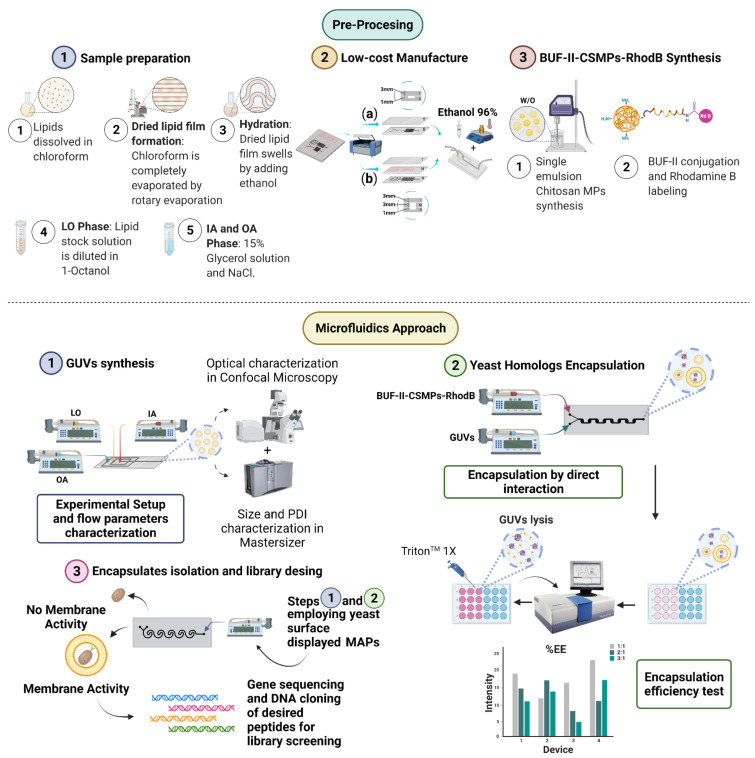
Schematic of Membrane-active Peptides (MAPs) library screening workflow. Pre-Processing stage steps: (1) Sample preparation of inner and outer aqueous phase and dried lipid film hydration for lipid-carrying organic phase preparation, (2) Low-cost laser cutting-based manufacturing technique for two- and three-layer devices, and (3) Synthesis of BUF-II-CSMPs-RhodB conjugates. Microfluidics approach steps: (1) Giant Unilamellar Vesicles (GUVs) synthesis based on octanol-assisted double emulsion templates, (2) Yeast homologues encapsulation by mixing-controlled direct interaction, finally (3) Encapsulates isolation and MAPs non-rational library design.

We have recently proposed the screening of a non-rational library of MAPs expressed on the surface of the yeasts *Kluyveromyces Lactis* and *Saccharomyces Cerevisiae* by a low-cost micromixer platform that allows their direct interaction with GUVs (Figure 1). In a pre-processing stage, the GUVs synthesis proceeded with alcohol as the lipid-carrying organic phase following the approach by Deshpande et al. within a microfluidic device [55]. Different flow conditions and sample preparations were evaluated to optimize the droplet generation and obtain cell-sized uniform and stable liposomes. These experiments were first conducted in silico via multiphysics simulations and confirmed experimentally employing devices manufactured in polymethyl methacrylate via laser cutting. In parallel, chitosan microparticles (CSMPs) were synthesized by a single emulsion technique and subsequently functionalized with the translocating peptide Buforin-II (BUF-II) as yeast homologues for a proof-of-concept screening experiment of positive cell penetration. Negative control of bare microparticles was included in the experimental set for comparison. BUF-II was fluorescently labeled with Rhodamine B (BUF-II-CSMPs-RhodB) to estimate the encapsulation efficiency achieved after intimate contact with GUVs via spectrofluorimetry (Figure 1—Pre-Processing). Encapsulation took place within passive micromixers with different channel geometries (Figure 1—Microfluidics Approach). The performance of the devices used for the GUVs synthesis was first explored in silico and then evaluated experimentally. Regarding the micromixers designed for encapsulation, the maximum efficiencies attained approached about 65%, which confirmed their suitability for the screening of non-rational libraries of MAPs expressed on yeasts surfaces. The performance was explained considering mixing efficiencies and the required trajectory lengths to reach them calculated both in silico and experimentally. Further engineering of the best performing devices will be pursued as future work along with the evaluation of the impact of changes in pH, temperature, ionic strength, and the presence of salts on the encapsulation performance. 

## 2. Materials and Methods

### 2.1. Materials

1-α-lecithin, soybean-cas 8002-43-5-calbiochem, and Chloroform c2432 > 99.5% were purchased from Merck (St. Louis, MO, USA). 1-Octanol and ethanol (96%) were purchased from PanReac AppliChem (Chicago, IL, USA). Chitosan (CS) (>75–85% deacetylation), glutaraldehyde (25%), acetic acid, hexane (99%), Tween 80, mineral oil, N-hydroxysuccinimide (NHS) (98%), N-[3-dimethylamino-propyl]-N’-ethyl carbodiimide hydrochloride (EDC) (98%), dimethyl sulfoxide (DMSO) (99%), rhodamine B (>95%) and polyethylene glycol were purchased from Sigma-Aldrich (St. Louis, MO, USA) and Triton X-100 was purchased from Thermo Fisher (Waltham, MA, USA). Buforin II (BUF-II, TRSSRAGLQFPVGRVHRLLRK) was synthesized by Peptide Synthesis Facility at Pompeu Fabra University and GL Biochem Shanghai (Shanghai, China). Purification was performed by HPLC (>95%) and molecular weights were confirmed via mass spectrometry. Poly(methyl methacrylate) (PMMA) 3-mm thickness sheets and dichloromethane were purchased from local distributors (Bogotá, Colombia).

### 2.2. Microfluidic Systems Design and Multiphysics Simulations

All simulations presented in this article were developed in a virtual machine with 32 cores and 64 GB of memory with the aid of COMSOL Multiphysics^®^ software.

#### 2.2.1. Double Emulsion Template for GUV Synthesis

Based on the Water-in-Oil-in-Water (WOW) liposome synthesis and Octanol-assisted Liposome Assembly (OLA) method, different geometry modifications were simulated taking into consideration results obtained by Muijlwijk et al. [59] for droplets’ formation of different sizes. Importantly, the simulation proposed for WOW droplets will generate double emulsions, which may serve as templates for GUV [60] production by the rearrangement of the lipid layers by solvent de-wetting.

**Figure 2:**
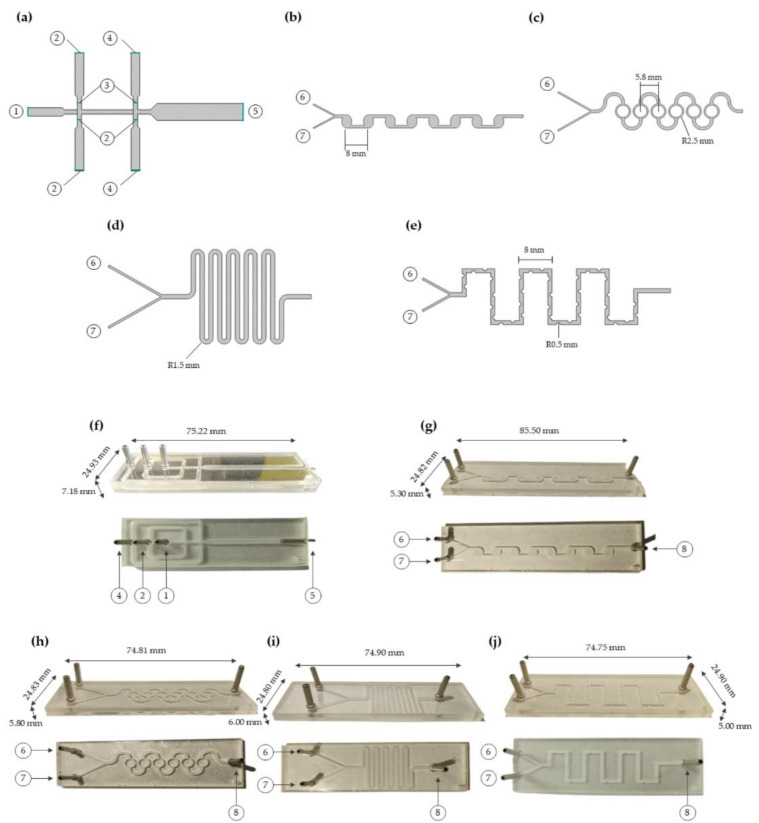
Design of 2D geometries for simulation of the microfluidic devices and further manufactured devices. (**a**) Double emulsion droplet generation microfluidic system. Inner continuous phase inlet of 1 mm width ①, dispersed phase inlet of 1 mm width ②, inlet channels constriction of 0.5 mm width and narrowing section junction ③. Outer continuous phase inlet of 1 mm width ④, and flow outlet channel of 2 mm width ⑤. (**b**) Chambers geometry micromixer. (**c**) SARS geometry micromixer. (**d**) Serpentine geometry micromixer. (**e**) Serpentine with circular features geometry micromixer and continuous (GUVs) ⑥ and dispersed (yeasts) ⑦ phase inlet for all micromixers, respectively. (**f**) WOW microfluidic system for the generation of double emulsions with their respective dimmensions, ① outer aqueous (OA) phase inlet, ② lipid-carrying organic (LO) phase inlet, ③ inner aqueous (IA) phase inlet, and ④ flow outlet. (**g**) Chambers, (**h**) SARS, (**i**) Serpentine, and (**j**) Serpentine with circular features micromixers with ⑤ GUVs inlet, ⑥ yeast homolgues (BUF-II-CSMPs-RhodB) inlet, ⑧ flow outlets and their respective dimensions.

Figure 2a shows the final 2D geometry proposed for the generation of WOW droplets based on the system developed by Campaña et al. for the generation of Alginate-Laccase microcapsules [61]. The system is comprised of two flow-focusing units [62], two inlet channels for the continuous phases (inner and outer aqueous phase represented as the center ①, and the second flow-focusing unit inlet channels ④ in Figure 2a) and an input channel for the dispersed phase (Figure 2a, ②). The input channels have 1 mm in width, and a 2 mm width output channel was modeled including a narrowing section of 0.5 mm width between flow-focusing units. The Two-Phase Flow, Level Set physics interface was implemented to study momentum transfer and double emulsion generation. The Level Set interface solves the incompressible formulation of the Navier-Stokes momentum Equation (1):

(1) $$\rho \frac{\partial u}{\partial t}+\rho \left(u\cdot \nabla \right)u=\nabla \cdot \left[-pI+\mu \left(\nabla u+\nabla u^{T}\right)\right]+F_{g}+F_{\mathrm{st}}+F_{\mathrm{ext}}+F$$

And the continuity Equation (2):

(2) ∇⋅u=0

where *ρ* is the density, **u** is the velocity vector, *p* is the pressure, **I** is the intensity vector, *μ* is the dynamic viscosity, **F_g_** is the gravity force vector, **F**_st_ is the surface tension force vector, **F**_ext_ is the user-defined volume force vector and **F** is the volume force vector.

The simulations were implemented for a Flow Rate Ratio (FRR) of 1:10:30 for the dispersed phase and continuous phases with initial velocities of 0.00185 m/s, 0.0185 m/s, and 0.0555 m/s, respectively. The rheological properties were those defined in COMSOL for the materials incorporated into the computational domain, i.e., water and vegetable oil for the continuous and dispersed phase, respectively. The no-slip condition was imposed on the walls and no pressure suppressing backflow was considered at the system’s outlet.

#### 2.2.2. Mixing-Induced Encapsulation by Direct Interaction

To induce interaction between the liposomes and the yeasts, different geometries of passive micromixers were proposed as shown in Figure 2b–e. All models were simulated using Mixture Model physics, which tracks the average phase concentration, or volume fraction, and solves a single momentum equation for the mixture velocity. The Mixture Model interface solves one set of Navier-Stokes Equation (3) for the momentum of the mixture defined as:

(3) $$\rho j_{t}+\rho \left(j\cdot \nabla \right)j+\rho_{c}\varepsilon \left(j_{\mathrm{slip}}\cdot \nabla \right)j=-\nabla p-\nabla \cdot \tau_{\mathrm{Gm}}+\rho g+F\;-\nabla \cdot \left[\rho_{c}\left(1-\phi_{c}\varepsilon \right)u_{\mathrm{slip}}j_{\mathrm{slip}}^{T}]-\rho_{c}\varepsilon [\left(j\cdot \nabla \right)j_{\mathrm{slip}}+\left(\nabla \cdot \left(D_{\mathrm{md}}\nabla \phi_{d}\right)-\frac{m_{\mathrm{dc}}}{\rho_{d}}\right)\right]$$

where **j** is the velocity vector, *ε* is the reduced density difference, **u**_slip_ is the slip velocity vector between the two phases, **j**_slip_ is the slip flux, *τ*_Gm_ is the sum of the viscous and turbulent stresses, *D*_md_ is a turbulent dispersion coefficient, *m*_dc_ is the mass transfer rate from the dispersed to the continuous phase, **g** is the gravity vector, and **F** is any additional volume force applied to the system.

The volume fraction of the dispersed phase is tracked by solving the transport Equation (4):

(4) $$\frac{\partial }{\partial t}\left(\phi_{d}\rho_{d}\right)+\nabla \cdot \left(\phi_{d}\rho_{d}u_{d}\right)=\nabla \cdot \left(\rho_{d}D_{\mathrm{md}}\nabla \phi_{d}\right)-m_{\mathrm{dc}}$$

where the continuous phase volume fraction is defined as *Φ*_c_ = 1 − *Φ*_d_.

The initial conditions established were a total flow rate (TFR) of 0.084 m/s with a FRR (liposomes:yeasts) of 1:1, 2:1, and 3:1 for the dispersed and continuous phase, respectively. The dispersed phase was defined as liquid droplets/bubbles. The dispersed fraction (Φ) was defined as 0.2 for inlet 1. Other parameters as rheological properties were established from the material employed over the computational domain (see Supplementary Materials Table S2). The no-slip boundary condition was imposed on the walls. The simulation was performed through a time-dependent study with a time interval of 15 s. As part of pre-processing work, convergence analysis was carried out for each geometry to guarantee mesh independent results (see Supplementary Materials Figure S2 for further information about details of the mesh convergence analysis). Finally, to avoid artificial diffusion and so, an erroneous estimation of molecular diffusion, steps reported from Bayareh [63] where taken into account to assure also a suitable mesh and robust computational results.

To determine the MRT and TL, a Particle Tracing Model was implemented. The calculation of additional degrees of freedom was conducted aided by auxiliary dependent variables. First-order ordinary differential equations (see Equations (5) and (6)) along each particle trajectory were solved with respect to time and position to determine MRT and TL, respectively.

(5) $$\frac{d}{dt}\left(rt\right)=1$$

(6) $$\frac{d}{ds}\left(tl\right)=1$$

where *rt* and *tl* are the residence time and the total length of the particle trajectory. For the same TFR and a FRR of 1:1, the time-dependent study was established with a time interval of 5 s. 50 particles were released at the beginning of the simulation and the MT, MRT, and TL were studied to ensure that the geometries and their mixing efficiency were comparable.

### 2.3. Device Fabrication and Manufacture

Low-cost manufacture of microfluidic systems was carried out based on the techniques presented in previous works by Bermudez et al., Campaña et al., and Aranguren et al. [61,64,65] and are summarized in Figure 1 (Pre-Processing—Low-cost manufacture). Masks were designed with the aid of Inventor^®^ (Autodesk Inc., Mill Valley, CA, USA) where red and black colors were used to identify zones for cutting and engraving, respectively. Laser cutting (TROTEC^®^ Speedy 100, 60 W, Marchtrenk, Upper Austria, Austria) and PMMA 2.5-mm width rectangular sheets were used for the fabrication of microfluidic devices. The assembly of the PMMA sheets was carried out by aligning the different layers and by applying 96% ethanol on the surface of the substrate, followed by maintaining constant pressure on the assembled device in a hot plate at 110 ° C. Microfluidic systems for the synthesis of liposomes were manufactured by the three-layer technique, cutting the mask in the middle layer, while micromixers for yeast homologues encapsulation consider two-layer ones, where the bottom layer included the engraved microchannel patterns.

### 2.4. Low-Cost Octanol-Assisted Liposomes Assembly (OLA)

Concerning the synthesis of cell-sized liposomes, the OLA methodology was implemented based on the protocol proposed by Deshpande et al. with slight modifications [55].

#### 2.4.1. Sample Preparation

The devices manufactured are presented in Figure 2f–i, consisting of one inner aqueous phase (IA) channel, two lipid-carrying organic phase (LO) channels, and two outer aqueous phase (OA) channels (See Figure 1 Pre-Processing—Sample preparation). For both aqueous phases, i.e., IA and OA, 50 mL of a glycerol solution at a concentration of 15% (*v/v*) was prepared. This solution was added to stabilize the double-emulsion droplets while NaCl 0.05 mM is added to the OA. To prepare the lipidic solution, soy lecithin was dissolved in chloroform at 1.5% (*w/v*). The sample was evaporated under reduced pressure at 150 RPM and 45 °C for 60 min. Once chloroform is evaporated, a lipid thin film is formed and then is hydrated with ethanol (96%) at 150 RPM and 50 °C under atmospheric pressure for 45 min. The final solution was sonicated and further homogenized by vigorous vortexing. Finally, the LO phase was prepared by dissolving the lipid solution on 1-Octanol at a concentration of 0.01%, 0.02%, and 0.04% (*v/v*).

#### 2.4.2. Experimental Setup

Four pieces of tubing of about 20 cm length (Nelaton, Probes, Medex caliber 8) were cut and connected to the solutions’ inlets and the outlet of the system. One 10 mL and two 20 mL syringes were loaded with the IA, LO, and OA phases, respectively, and securely mounted on syringe pumps (78-8110C Programmable Touch Screen, Cole-Parmer^®^, Holliston, MA, USA, and MP-30 Syringe Pump, MedCaptain). Flow conditions were set to maintain FRR values of 1:10:30, 1:20:60 and 1:30:90 (IA:LO:OA) for TFR values of 410 mL/h, 810 mL/h and 810 mL/h, respectively. A schematic of the experimental setup is shown in Figure 1 (Microfluidics approach—GUVs synthesis). The obtained solutions were collected by locating the outlet tubing into a reservoir recipient after each solution had entered the device and the flow was stabilized.

### 2.5. GUVs Characterization

GUVs size distributions were measured via static light scattering (SLS) using a Mastersizer 3000 Hydro MV unit (Malvern Panalytical, Malvern, Worcestershire, UK) by considering the refractive and absorption indexes of soy lecithin i.e., 1.46 and 0.001, respectively. Volume-weighted average diameter (d_43_) was regarded as the mean diameter for all measurements. Morphology and size of GUVs were evaluated by collecting images in an Olympus FV1000 Confocal Laser Scanning Microscope (CLSM) (Olympus, Shinjuku, Tokyo, Japan) with a PlanApo 20× and 40×, 1.2 NA objective.

To perform data analysis, the GraphPad Prism Software (GraphPad Software, La Jolla, CA, USA) was used. A Two-way ANOVA was implemented to make statistical comparisons. The factors evaluated were the FRR and the concentration of LO phase. The results with *p* ≤ 0.05 (*) were deemed significant. Data are presented as average ± one standard deviation.

### 2.6. Synthesis of BUF-II-CSMPs Conjugates and Labeling with Rhodamine B

Synthesis of chitosan microparticles was conducted by following the method reported by Mothilal et al. with slight modifications [66]. Briefly, the aqueous phase was prepared by dissolving 2% (*w/v*) chitosan in a solution of 4% (*v/v*) acetic acid under magnetic stirring at 500 RPM for 24 h. The oil phase consisted of mineral oil and Tween 80 at a concentration of 2% (*v/v*). A W/O emulsion was formed by adding 5 mL of the aqueous phase with a 22G syringe into 100 mL of the oil phase and stirred for 10 min at 600 RPM aided by a mechanical stirrer Hei-TORQUE Precision 200 (Heidolph, Schwabach, Germany). Subsequently, 1 mL of glutaraldehyde solution was added and kept under stirring for 2 h at 300 RPM. Separation of the microparticles was achieved by centrifugation at 3600 RPM for 10 min followed by thoroughly washing with hexane and type II water (water with a resistivity >1 MΩ-cm, and conductivity <1 µS/cm) thrice. 

**Figure 3:**
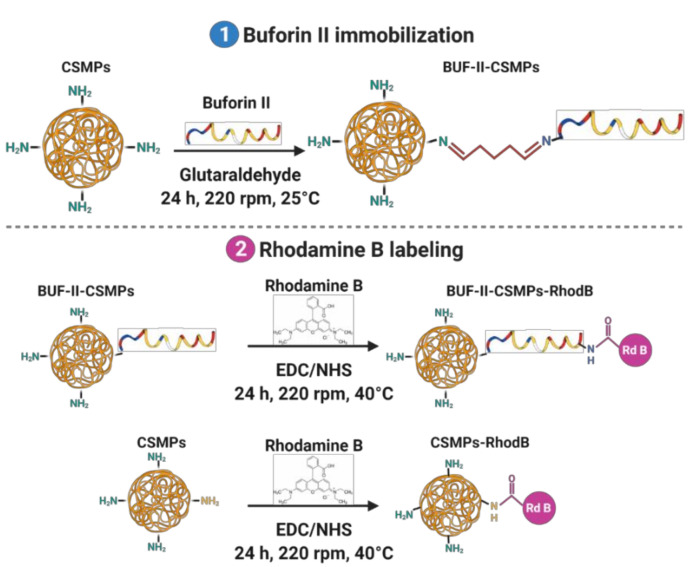
Schematic of the functionalization and labeling of the MPs: (1) Buforin II conjugation by forming imine bonds between its N-terminal and the pendant amine groups on the CSMPs surface mediated by glutaraldehyde as crosslinking agent. (2) Labeling with Rodamine B by conjugation to immobilized BUF-II and bare CSMPs through amide bonds formed aided by EDC/NHS.

To obtain the Buforin II conjugated chitosan microparticles (BUF-II-CSMPs, see Figure 3 step 1), 100 mg of CSMPs were suspended in 50 mL of type II water and sonicated in an ultrasonic bath for 10 min. BUF-II was conjugated by its N-terminal to the amine groups in the CSMPs with glutaraldehyde. Briefly, 2 mL of glutaraldehyde 2% (*v/v*) was added to the CSMPs suspension and kept under magnetic agitation for 1 h. Then, 1 mg of the peptide in 5 mL of type I water (Ultrapure water with a resistivity >18 MΩ-cm, and conductivity <0.056 µS/cm) is added and kept under stirring at 220 RPM for 24 h. After conjugation, samples were thoroughly washed with type II water by centrifugation at 3600 RPM to remove excess reagents. For the labeling with Rhodamine B, 14 mg of EDC and 7 mg of NHS were well mixed in 10 mL of type II water. Then 5 mg of Rhodamine B and 2 mL of DMF 50% (*v/v*) were added to the solution. The mixture is heated up to 40 °C under continuous magnetic agitation for 15 min. This allows activation of the Carboxyl groups of Rhodamine B to subsequently form amide bonds with the free amine groups of the BUF-II. Finally, the mixture was added in the BUF-II-CSMPs suspended in 50 mL of type II water and left to react for 24 h under continuous agitation at 220 RPM [67]. After conjugation, Rhodamine B labeled with Buforin II chitosan microparticles (BUF-II-CSMPs-RhodB, see Figure 3 step 2) were thoroughly washed with type II water and centrifuged at 3600 RPM to remove excess reagents. The microparticles labeled with Rhodamine B (CSMPs-RhodB, see Figure 3 step 2) were prepared also as described above. CSMPs and bioconjugates were stored at 3 °C until further use.

### 2.7. Characterization of CSMPs Bioconjugates and Encapsulates

Microscopic inspection of morphology, size, and shape of the CSMPS was done with a Scanning Electron Microscopy (SEM) in a JSM 6490-LV TESCAN (JEOL, Tokyo, Japan) at 600× and 400× magnifications and a 10 kV accelerating voltage. Sequential surface modifications of CSMPs with Rhodamine B and BUF-II were evaluated via Fourier Transform Infrared Spectroscopy (FTIR) by a Bruker Alpha II FTIR Eco-ATR (Bruker, Billerica, MA, USA). Spectra were collected in the range of 4.000–600 cm^−1^ with a spectral resolution of 2 cm^−1^. Thermogravimetric analysis (TGA) was carried out by ramping up the temperature of an 8 mg sample at a rate of 10 °C/min from 30 to 600 °C in a simultaneous TGA/DSC instrument (TA Instruments, New Castle, DE, USA). Imaging of BUF-II-CSMPs-RhodB and encapsulates was conducted on an Olympus FV1000 confocal laser scanning microscope (CLSM) (Olympus, Shinjuku, Tokyo, Japan) with a PlanApo 20× and 40×, 1.2 NA objective. The samples were imaged by exciting the samples with the instrument’s 546 nm laser.

### 2.8. Colorimetric Mixing Efficiency Test

A colorimetric mixing efficiency test was conducted to evaluate mixing quality [68]. A solution of NaOH 1M and phenolphthalein 0.1% (*w/v*), as pH indicator, were pumped at a controlled TFR of 150 mL/h and a FRR of 1:1. The liquids were initially colorless but upon mixing, the color will gradually change to violet red. Based on the standard deviation of the pixel intensity, the mixing quality was measured by Equation (7).

(7) $$\sigma =1-\sqrt{\frac{1}{N}\sum_{i=1}^{N}\left(\frac{I_{i}-I_{\mathrm{mix}}}{I_{\mathrm{unmix}}-I_{\mathrm{mix}}}\right)}$$

where $I_{i}$ is the pixel intensity in the mixing picture, $I_{\mathrm{unmix}}$ is the pixel intensity before mixing and $I_{\mathrm{mix}}\;$is the pixel intensity after complete mixing and *σ* ranges from 0 for non-mixing to 1 for homogeneous mixing. Images were processed computationally, and the mixing efficiency was then calculated with a Python script (Supplementary Materials Script S1).

### 2.9. Encapsulation of CSMPs Bioconjugates as Homologues of MAPs 

Although using microfluidic devices generally leads to saving reagents due to the small amount volumes involved, as for bulk experimentation techniques, it is advisable to carry out proof-of-concept experiments to evaluate the devices’ performance before implementing experiments possibly involving difficult-to-prepare and expensive reagents. In this regard, we synthesized and purified BUF-II-CSMPs-RhodB of 5–10 µm as homologues of yeasts expressing MAPs on their surfaces to evaluate both the impact of changing the geometry of the device and key operating parameters (e.g., FRR and TFR) on the mixing efficiency and consequently the encapsulation probability. This was with the idea of selecting the device with the highest performance for further future testing with the yeasts expressing the non-rational library of MAPs. A parametric sweep was implemented for different FRR and TFR values. The encapsulation efficiency was evaluated by fluorescence intensity changes before and after treatment with the lipid bilayer destabilizing detergent Triton X-100. For this characterization, 100 µL of encapsulates (BUF-II-CSMPs-RhodB-GUVs) was pipetted into a 96-well microplate for the first fluorescence emission measurement. 20 µL of Triton X-100 was then added quickly to each sample to assure disruption of the GUVs membrane and the subsequent release of BUF-II-CSMPs-RhodB. Subsequently, a second fluorescence intensity measurement was then carried out to monitor possible changes with respect to the untreated sample. A Spectrofluorometer (0239D-2219 FluoroMax plus C, Horiba, Miyanohihashi, Japan) was set up with excitation at 546 nm and emission intensity measurement at 568 nm, which were selected according to the fluorescence spectrum of Rhodamine B. Encapsulation efficiency was estimated by the Equation (8) below.

(8) $$\%\;EE=\frac{I_{\mathrm{pt}}-I_{\mathrm{bt}}}{I_{\mathrm{pt}}}\cdot 100$$

where *I*_pt_ and *I*_bt_ refer to the fluorescence reading obtained post- and before-Triton X-100 treatment, respectively.

Finally, negative vs. positive control test was developed evaluating %EE of CSMPs-RhodB within the same conditions of the positive control explained above. Data analysis was conducted using the paired t test for statistical comparisons. The results were considered significant if they have a *p*-value less than 0.05 (*). The data is shown as mean ± standard deviation.

## 3. Results and Discussion

### 3.1. Multiphysics Simulations

#### 3.1.1. Double Emulsion Generation

**Figure 4:**
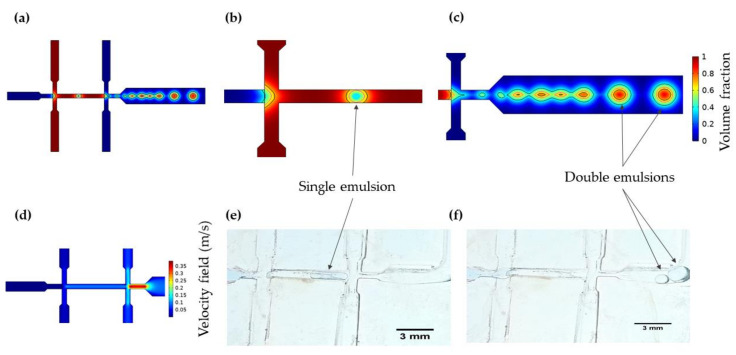
Simulation results for double emulsion generation and experimental visualization via digital microscopy. (**a**) Dispersed phase volume fraction in two-step double emulsion formation. (**b**) Single emulsion formation within the first flow-focusing unit with contours indicating inner continuous phase (black) surrounded by dispersed phase (red domain). (**c**) Double emulsion formation at the second flow-focusing unit with contours indication inner aqueous phase (black) and dispersed phase (magenta) surrounded by outer continuous phase (blue domain). (**d**) Velocity profile for a 1:10:30 FRR. (**e**) Experimental single and (**f**) double emulsion formation with the assembled microfluidic system.

The simulation results shown in Figure 4a allowed direct observation of double emulsion droplets forming within the WOW geometry. Figure 4b,c show the breakup of the inner aqueous phase into a single emulsion and the subsequent formation of a double emulsion as well as the contours that delineate the continuous and dispersed phases. In the single emulsion formation, it was evidenced that the droplet generation is governed by a dripping regime where drops are formed near the first flow-focusing unit junction. In this case, inertial force is largely negligible while the interfacial force is dominant and consequently, droplet formation begins with an increase of viscous force over the pinning force [69]. At the second flow-focusing unit, double emulsion formation is achieved within a jetting regime where a long jet that further breaks into droplets in the outlet channel is visualized using a two-step emulsification method [70]. This regime exists when the outer phase shearing force dominates and exceeds the inner phase shearing force [69]. Because droplet size is related to the size of the emulsions [71], we evaluated the impact of varying the TFR and the FRR on the size of the droplets, and the rate of droplet generation. The results showed that by increasing the velocity of the continuous phase, the droplet size is reduced. Likewise, increasing the velocity of the dispersed phase increases the droplets’ generation rate. This can be explained by the relation between viscous forces and the interfacial tension of the phases, which can be understood by looking at the capillary number (Ca) (see Equation (9) below).

(9) $$Ca=\frac{\mu \cdot u}{\sigma }$$

According to Conchouso et al., high Ca increases shearing forces, which result in smaller droplets [72]. Based on this, a direct and inverse relationship was identified between FRR and TFR and the droplet generation mechanism, respectively. Furthermore, simulations showed a constant velocity profile for the inlets in the first flow-focusing unit with a slight increase in the narrowing section before the second unit, which is in agreement with previous reports [61]. Likewise, a significant increment in the continuous phase velocity is achieved at the constriction of the double emulsion generation nozzle, but the pressure drop is not enough to maintain the dripping regime because of the resistance offered by the single emulsion [70]. However, the added constriction minimizes the surface tension energy, contributing to generating large and stable droplets at a good rate (Figure 4c) [72]. Finally, the mesh convergence study showed that above 1112 elements, variations in the velocity magnitude are below 4%. Also, the sensitivity analysis demonstrated a significant impact of the TFR on the model’s results, unlike the subtle changes evidenced when evaluating the FRR (See Supplementary Materials Figure S1).

#### 3.1.2. Encapsulation by Direct Interaction

The obtained in silico results and the analysis for the three evaluated FRR can be seen in detail in the Supplementary Materials (Figure S3). In general, the calculated velocity profiles exhibit similar behavior, reaching a maximum velocity at the center of the micromixers. To evaluate the mixture, the volume fraction of the dispersed phase (ϕ_d_) was tracked along the mixer length at a time where the mixture reached complete homogeneity. As the FRR went from 1:1, to 2:1 and 3:1 the ϕ_d_ showed an increment, but the main behavior remains unchanged. These results strongly suggest that the liposomes phase (ϕ_d_) will increase in the mixture along with the FRR’s change.

**Figure 5:**
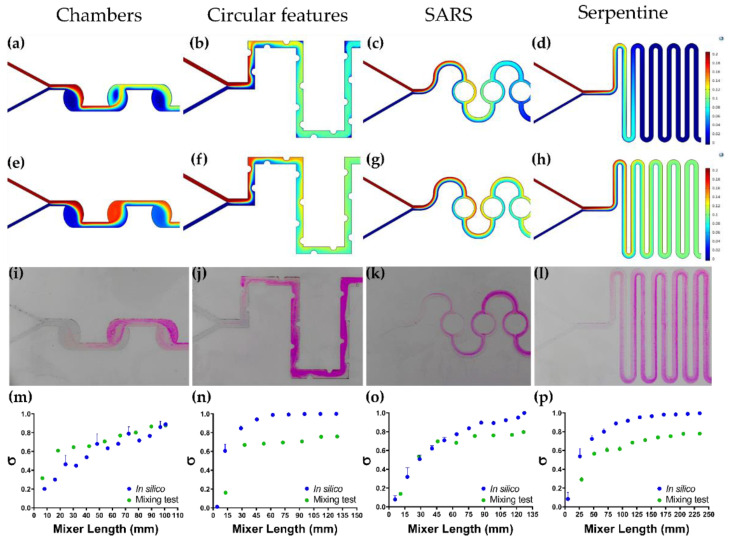
Dispersed phase volume fraction simulations and colorimetric mixing efficiency results in silico and in vitro. (**a**) Chambers, (**b**) Serpentine with circular features, (**c**) SARS, and (**d**) Serpentine mixing profile (ϕ_d_) at 2 s. (**e**) Chambers, (**f**) Serpentine with circular features, (**g**) SARS, and (**h**) Serpentine mixing profile (ϕ_d_) at 15 s. (**i**) Chambers, (**j**) Serpentine with circular features, (**k**) SARS, and (**l**) Serpentine in vitro colorimetric mixing profile. (**m**) Chambers, (**n**) Serpentine with circular features, (**o**) SARS, and (**p**) Serpentine in silico and in vitro mixing efficiency along micromixer length.

For a fair comparison of the micromixers’ performance, MT, MRT, and TL were calculated (Table 1). First, the circular chambers’ geometry (Figure 5a,e) showed a highly homogeneous mixing with an efficient of 88.83 ± 1.76% in silico and 87.60 ± 0.53% in vitro, and a ratio of mixing index to pressure drop (ME/ΔP) of 2.94 ± 0.08, with a mixing time of approximately 12.76 ± 1.13 s. This performance could be explained by the ability of this micromixer to mix fluids by the formed self-circulating and chaotic flow streams within the chambers. Thus, narrower constriction channels increase mixing efficiency and pressure drop [73,74]. Also, a relevant factor in this device is the relatively large area of the circular chamber, which can improve even more the efficiency of the micromixer. Additionally, particles entering the chambers experience a circulating flow downstream from the inlet to the microchannel, which increases the residence time of the fluid within the micromixer and consequently, the mixing efficiency [72].

The serpentine channel with circular features demonstrated an efficient mixing of about 99.97 ± 0.02% in silico and 75.86 ± 0.12% in vitro (Figure 5b,f), and a ME/ΔP of 1.24 ± 0.03 with an estimated mixing time of 3.85 ± 0.15 s, which is about three times shorter than the previous micromixer. Taking into account the ME/ΔP, micromixers’ performance was between the four and five mixing cycle of the micromixer reported from [75]. This superior mixing efficiency can be related to the generation of vortices and chaotic advection induced by the circular features present in the geometry, which restrict flow, thereby leading to a higher pressure drop and a better fluid interaction [76,77,78]. Thus, it is expected that the constant collisions associated with chaotic advection might favor encapsulation, as this phenomenon occurs in the transverse direction of the microchannel and increases the interfacial area in an exponential matter, which, in turn, enhances mixing [74,79]. 

Next, SARS geometry (Figure 5c,g) showed a good mixing level within approximately 4.61 ± 0.70 s with an efficient of 95.05 ± 0.64% in silico and 79.70 ± 0.94% in vitro, and a ME/ΔP of 1.52 ± 0.16. The mixing process within SARS microchannels takes place by splitting the fluid at its junctions, where the two fluid streams travel through the circular channels and the resulting Dean effects concentrate the fluids’ components at their interface [68]. These Dean vortex flows give rise by the centrifugal effects generated due to the dislocation of the flow in the sub-channels [80]. In the same way, the velocities of the sub-streams may differ in magnitude and direction, therefore re-coalescence might take place, which is likely to cause fluid stretching and consequently an enhanced mixing [68,78]. In this manner, the recirculation of the flow and the flipping of the streamlines generates chaotic advection in the microchannels, which expands exponentially the interfacial area and diminishes the diffusion length, inducing an efficient homogenization of the fluids [79].

Lastly, the Serpentine channel geometry (Figure 5d,h) shows an efficient mixing between the continuous phase and the dispersed phase with a mixing time of approximately 7.95 ± 0.92 s and an efficient of 99.58 ± 0.13% in silico and 77.96 ± 1.49% in vitro, and a ME/ΔP of 1.71 ± 0.06. This can be attributed to the serpentine’s microchannel length, which allows the two phases to stay longer within the turns of the device [81,82]. This allows the streamlines to help to maintain a continuous phase interaction, favoring simple diffusion. This facilitated mixing is favored even further by induced secondary flows that can produce chaotic advection and a higher pressure loss, which, in turn, induces a stronger flow instability and the interaction of flow streamlines [68,83]. In consequence, mixing biomolecular streams through chaotic advection may reduce the damage to large molecules [78]. 

Finally, considering the results for the mixing time, a sensitivity analysis was carried out to evaluate the most significant parameters influencing mixing. To achieve this, FRR and TFR were varied by ± 20%, and the volume fraction of the dispersed phase was evaluated until a steady state is reached at a point located at about 75% of the characteristic length of the system. The results show that the most sensitive parameter was FRR as evidenced by a directly proportional impact on the volume fraction of the dispersed phase (Supplementary Materials, Figure S5).

### 3.2. Low-Cost Octanol-Assisted Liposomes Assembly

According to the digital microscopy images presented in Figure 4e,f, the single emulsion is formed at the first flow-focusing unit while the double one is formed in the second flow-focusing unit after splitting the single emulsion into two droplets. The double emulsion was successfully formed in a two-step process where two dripping pressure-based instabilities take place in each flow-focusing unit, producing an inner aqueous phase droplet contained within a lipid-carrying organic phase outer droplet [84,85]. Depending on the FRR, different IA:LO ratios might be achieved, which have a significant impact on defining the droplet generation regime. In this regard, higher IA:LO ratios lead to a dripping regime, while lower ones produce a pseudo-jetting regime where elongated droplets with weaker surface tension are formed. For all the evaluated FRRs (see Supplementary Materials, Table S3) a dripping regime is largely predominant, which is likely to translate into forming monodisperse droplets [85]. In addition, droplet formation within the device was verified by direct microscopic visualization, which indicates that the generated pressures are enough to produce inner and middle phases break up into these structures, as predicted by the in silico analyses (See Supplementary Materials, Video S1, Video S2, Video S3). Concerning octanol de-wetting and separation on-chip demonstrated by Deshpande et al. [86], LO phase concentration was adjusted to provide an appropriate interfacial tension that induces spontaneously de-wetting. This assures the separation of octanol residues from GUVs in a two-phase sedimentation process that takes about 1–2 h. This principle of separation by sedimentation is due to the octanol buoyancy that allows its precipitation for further supernatant extraction off-chip [87], which makes the proposed device a simpler low-cost alternative with high effectiveness for the synthesis of GUVs.

### 3.3. GUVs Characterization

**Figure 6:**
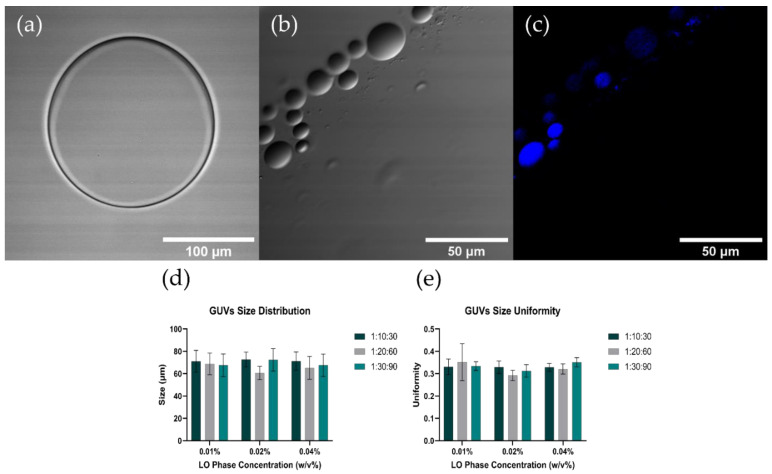
GUVs size and shape characterization. (**a**–**c**) CLSM images of GUVs. (**d**) SLS GUVs size distribution and (**e**) uniformity characterization.

Figure 6a–c show the CLSM characterization of the synthesized GUVs. The images showed correctly formed GUVs with a lipid bilayer and encapsulated methylene blue inner aqueous medium. The images also showed that the sizes of the GUVs obtained are in the range of 5–150 µm, which agrees well with the average sizes and uniformity obtained by SLS (Figure 6e,f). For all the evaluated configurations, the obtained GUVs presented an average size of around 60–80 µm. As shown in Figure 6e, for a LO phase concentration of 0.01% there is a slight decrease with no statistical significance in the size when increasing the FRR. This is closely linked to the inner flow rate and the IA:LO ratio, whereby by increasing the FRR an increase in the difference between center and side-channel inflows is observed. This generates smaller and more compact simple emulsions, leading to GUVs of lower size [61,85]. This relationship is no longer applicable at higher concentrations (0.02% and 0.04%) as evidenced by the increase in size obtained for higher FRR and the results obtained in the statistical comparisons. This can be explained from the dimensions of the channels of the manufactured device, which are crucial to maintain a laminar regime within the microsystem and to have finer control over the size of the formed emulsions [61]. The uniformity of the synthesized GUVs was under 0.4 for all evaluated operating conditions, indicating that monodispersity was preserved to an acceptable degree as expected for two-step double emulsion generators [85]. Finally, there is no apparent relationship between the LO phase concentration and the GUVs size.

### 3.4. CSMPs and Bionconjugates Characterization

**Figure 7:**
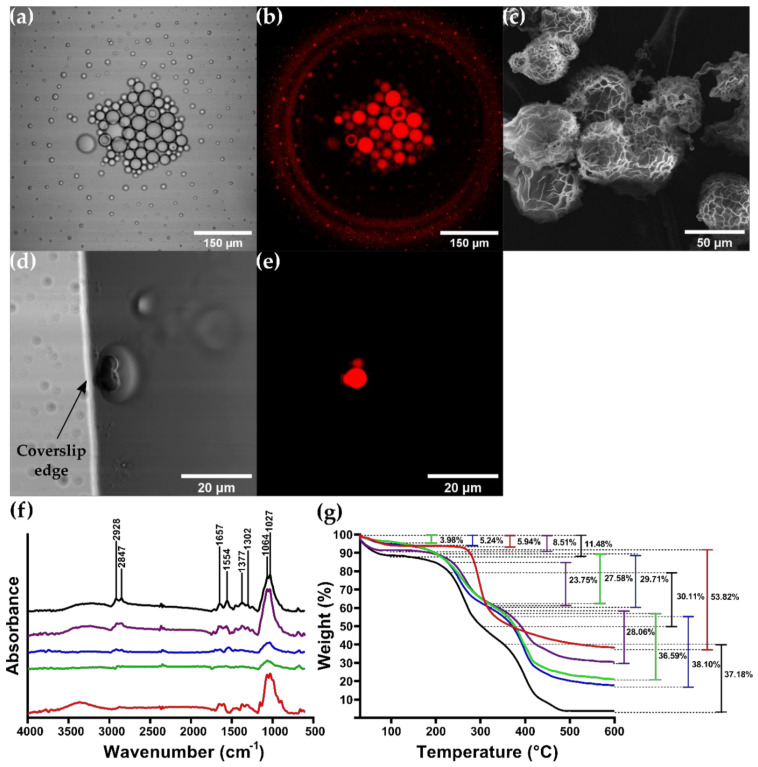
CSMPs size, shape and morphology characterization and encapsultes observation. (**a**,**b**) CLSM images of BUF-II-CSMPs-RhodB confirm micro size and sucessful labeling, respectively. (**c**) SEM image of CSMPs. (**d**,**e**) CLSM images of BUF-II-CSMPs-RhodB—GUVs encapsulates. (**f**) FTIR spectra of CS (red), CSMPs (green), BUF-II-CSMPs (blue), CSMPs-RhodB (purple) and BUF-II-CSMPs-RhodB (black) confirm surface modifications and main peaks of imine bonds (1554 cm^−1^) and amide I bands (1650 cm^−1^). (**g**) TGA thermograms of CS (red), CSMPs (green), BUF-II-CSMPs (blue), CSMPs-RhodB (purple) and BUF-II-CSMPs-RhodB (black) showed a first weight loss step (3.98 to 11.48%) that represents the dehydration of the samples. A second weight loss step (23.75 to 53.82%) due to the depolymerization of chitosan backbone chain and the degradation of the glycosidic bond in the case of CS. The final weight loss step (28.06 to 37.18%) is attributed to the degradation of glutaraldehyde and detachment of BUF-II and Rhod-B from the MPs surface.

Scanning electron microscopy (SEM) was implemented to visualize the surface morphology and size of the synthesized CSMPs (Figure 7c). The images revealed that the CSMPs exhibit important polydispersity with a size distribution ranging from 2–40 μm. Also, they show a round yet elongated flower-type morphology as evidenced by the roughness of the surface. This has been observed previously for emulsion syntheses methods due to the impact of varying the chitosan concentration and the crosslinking conditions [88,89,90]. Figure 7a,b show the confocal laser scanning micrographs (CLSM) of BUF-II-CSMPs-RhodB. The images confirm the polydispersity and circular shape of the microparticles obtained, as well as the adequate labeling of BUF-II-CSMPs with Rhodamine B, through the fluorescent emission of BUF-II-CSMPs-RhodB [91,92]. Fourier transformed infrared spectroscopy (FTIR) and thermogravimetric analysis (TGA) were used to confirm surface modifications and thermal stability of the CSMPs. Figure 7f shows the FTIR spectra of the CS, CSMPs, CSMPs-RhodB, BUF-II-CSMPs (before labeling with Rhodamine B), and BUF-II-CSMPs- RhodB (after labeling with Rhodamine B). The spectrum of CS showed a band between 2850 to 2919 cm^−1^, which has been typically associated with the stretching vibration of C-H groups [93]. The stretching C=O group at 1649 cm^−1^ can be attributed to the acetamide group present in chitosan [93,94]. Bands at 1372 cm^−1^ and 1298 cm^−1^ correspond to the stretching vibration of the C-N bond and bending vibration of C-H, respectively [93]. A band at 1554 cm^−1^ was identified for CS, CSMPs, BUF-II-CSMPs, CSMPs-RhodB, and BUF-II-CSMPs-RhodB spectra, which represent the C=N of imine bonds formed between amine groups of CS and carbonyl groups of glutaraldehyde through a Schiff base reaction [93]. Also, a band at around 1650 cm^−1^ was observed in BUF-II-CSMPs, CSMPs-RhodB, and BUF-II-CSMPs-RhodB, which can be assigned to the amide I band, a C-O stretching mode together with an N-H deformation mode [67,94,95]. 

CSMPs thermal stability and extent of surface functionalizations in bioconjugates were also estimated via TGA (Figure 7g). Pure CS exhibited two weight loss stages, while bare CSMPs and bioconjugates exhibited three. An initial weight loss of 5.94% for CS, 3.98% for CSMPs, 5.24% for BUF-II-CSMPs, 8.51% for CSMPs- RhodB, and 11.48% for BUF-II-CSMPs-RhodB in the range of 30 °C to 100 °C was associated with a loss of adsorbed/bound water/moisture vaporization [96]. The second weight loss of 53.82% observed in CS was in the range from 264.71 °C to 348.30 °C, which is attributed to the degradation of the glycosidic bond of chitosan [93]. For the CSMPs and BUF-II-CSMPs, the second weight losses between 193.21 °C and 315.77 °C were 27.58% and 29.71%, respectively. CSMPs-RhodB degrades from 235.39 °C to 329.23 °C with a weight loss of 23.75% and BUF-II-CSMPs-RhodB degrade from 222.34 °C to 297.54 °C with a weight loss of 30.11% mainly due to depolymerization of the chitosan backbone chain [93]. In the final weight-loss stage, the degradation range was from 348.09 °C to 406.04 °C for CSMPs, BUF-II-CSMPs, and CSMPs-RhodB with a final weight loss of 36.59%, 38.10%, and 28.09%, respectively. For BUF-II-CSMPs-RhodB, the degradation range was from 348.09 °C to 422.11 °C with a final weight loss of 37.18%, due to degradation of glutaraldehyde as well as the detachment of conjugated Buforin-II or Rhodamine B [67,93,95].

### 3.5. Colorimetric Mixing Efficiency Test vs. Mixing Efficiency In Silico

The experimental results for each micromixer and the results of the mixing efficiency for the in silico and the experimental approaches can be observed in Figure 5. In general, both computational and experimental results show a remarkable agreement for all studied micromixers, where during the initial contact section mixing is increasingly dynamic but stabilizes as soon as the mixture reaches homogeneity. Hence, the sooner the mixture becomes stable, the higher the chances to obtain a superior mixing efficiency. Conversely, the serpentine micromixer equipped with circular features is the best one as it stabilizes after the mixture travels some 30 mm length (Figure 5n). This is followed by the serpentine (Figure 5p), and SARS (Figure 5o), where it takes about 50 mm and 65 mm, respectively. Finally, the chambers geometry showed the worst performance (Figure 5m) as stability was never fully reached for the studied microchannel length. Besides traveled distances to reach stability, a full assessment of efficiency requires estimating the level of mixing achieved. In this regard, a comparison of in silico and experimental mixing levels is shown in Figure S8. On the one hand, Figure S8a summarizes the results for the mixing levels obtained in silico for all the evaluated micromixers. The highest mixing level was obtained at a length of about 90 mm with the serpentine channel with circular features micromixer. This was followed by the SARS, serpentine, and chambers geometries micromixers. As discussed above, the serpentine channel with circular features generates chaotic advection within the microchannels, which improves the mixing process considerably. On the other hand, the results of the colorimetric mixing efficiency tests for all the evaluated micromixers are shown in Figure S8b. Contrary to what was observed in silico, the micromixer reaching the highest mixing level at a channel length of around 90 mm was chambers. This was followed by SARS, serpentine with circular features, and serpentine microchannels. This superior performance might be possibly related to the induced self-circulating flows within the channels of the chambers micromixer.

### 3.6. Encapsulation Bioconjugate Homologues

**Figure 8:**
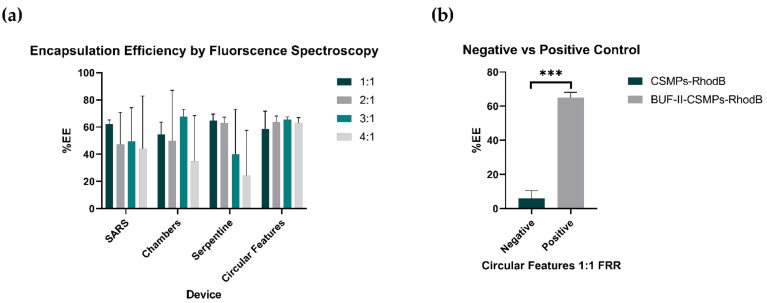
Encapsulation efficiency results for the (**a**) SARS, Chambers, Serpentine, and Serpentine with circular features micromixers at FRRs of 1:1, 2:1, 3:1, and 4:1. (**b**) Negative vs. Positive control test results for the encapsulation efficiency of CSMPs-RhodB and BUF-II-CSMPs-RhodB using the Circular Features device at 1:1 FRR.

The encapsulation efficiencies (%EE) of BUF-II-CSMPs-RhodB homologues into GUVs was determined by measuring the fluorescence emitted by a sample before and after applying the detergent Triton 100-X. The results for different operating FRRs are shown in Figure 8. Except for the circular features geometry, the FRR appears to impact encapsulation efficiency for all the evaluated micromixers. However, this trend is not significant (ns) according to the results of the statistical analysis conducted. The average %EE attained with the circular features micromixer was above 60% for all the evaluated FRRs, even reaching values close to 65% for 2:1, 3:1, and 4:1 FRR. Additionally, this device exhibited the lowest variability of all between replicates. This agrees well with the results obtained for the mixing efficiency tests carried out both computationally and experimentally, and the negative vs. positive control test where non-penetrating MPs (CSMPs-RhodB) were unable to internalize the GUVs so their %EE was significantly (***) low as shown in Figure 8b. This results demonstrates its potential for conducting the screening of MAPs libraries expressed on yeast surfaces, which is in agreement with similar previous works [97,98]. Despite the attractive results, further experiments will be needed to evaluate the impact of other operating conditions including, different pH values, ionic strengths, and the presence of salts on the screening efficiency of MAPs [49].Regarding Serpentine and Chambers geometries, the 1:1 and 2:1, and 3:1 FRRs led to average %EE in the range of 62.9–67.6%. The SARS micromixer showed the lowest average %EE for the evaluated FRRs. Due to statistical non-significance, their potential as devices for the screening of MAPs remains under study. Despite their acceptable performance, it is worth mentioning that the devices showed high variability between replicates, which might be disadvantageous during the MAPs screening. This can be explained by the limited chances to achieve encapsulation by direct interaction as immobilized MAPs (or expressed on yeasts surfaces) require a considerably long interaction time to intermingle with lipid bilayers prior to translocating them. This has been addressed previously by encapsulating MAPs into double emulsions as this ensures longer interaction times [99]. 

Overall, GUV bulk studies lack control and suffer from the need of sacrificing spatiotemporal resolution for throughput or vice versa [100]. When it comes to microfluidics, the majority of methods rely on vesicle trapping within a microchannel to allow total exchange of the vesicle’s surrounding fluids for subsequent MAPs analysis [100]. This inevitably leads to more complicated processing, complementary techniques for visualization and evaluation, and the need for implementing lipid modifications that may detrimentally affect membrane properties [100]. The framework proposed here overcomes these limitations by considering MAPs screening as the encapsulation of a bioactive compound after constant and successive direct interactions with the lipid bilayer of giant liposomes (GUVs) in a two-phase mixture context. Therefore, the promising results demonstrated by the circular features micromixer in terms of encapsulation efficiency makes it appealing for further engineering focused on finetuning the operating parameters and extending RT and TL such that a more efficient mixing might be attainable.

## 4. Conclusions

The screening of MAPs can be accelerated by a low-cost and robust methodology based on the encapsulation of yeast surface-displayed MAPs homologues (i.e., the membrane-active peptide Buforin II (BUF-II) conjugated to chitosan microparticles (CSMPs)) by the controlled and direct interaction with GUVs that mimic the composition of cell membranes. Synthesis of large and stable GUVs was conducted by forming double emulsions within low-cost microfluidic devices by adapting the octanol-assisted, on-chip de-wetting method, which avoids the use of sophisticated equipment, or specialized facilities such as clean rooms. Prior to manufacture, the device performance was explored in silico aided by Multiphysics simulations, which allowed us to identify operating conditions leading to droplet sizes in the range of 5–150 µm. The validity of the simulations was successfully confirmed experimentally. CSMPs were successfully synthesized, functionalized, and characterized spectroscopically (FTIR), thermally (TGA), and microscopically (CLSM). 

We proposed to achieve the controlled interactions between GUVs and BUF-II-CSMPs with the aid of passive micromixers. We explored the impact of different microchannel geometries and operating conditions on the mixing level achieved (and consequently encapsulation efficiency) for specific residence times and characteristic lengths via Multiphysics simulations. Encapsulation efficiencies were also measured experimentally using fluorescently labeled BUF-II-CSMPs conjugates and demonstrated reasonable agreement with simulations. A serpentine micromixer equipped with circular features showed the highest average encapsulation efficiencies, approaching about 65% for the 2:1, 3:1, and 4:1 flow rate ratios (FRRs). This was explained by the unique mixing patterns achieved within the device, which emerge due to the chaotic advection induced by the circular features present in the geometry, which restrict flow, thereby leading to a higher pressure drop and a better interaction between the interacting components. Our findings show that passive micromixers provide a suitable route for the screening of non-rational libraries of MAPs expressed on yeasts surfaces and open the possibility for further device engineering to enhance mixing levels and to explore other conditions that are critical for performance including, changes in pH, ionic strength, and presence of salts.

## Figures and Tables

**Table 1:** Micromixing parameters. Mixing time (MT), mean residence time (MRT), trajectory length (TL) (Characteristic length), pressure drop (ΔP), mixing efficiency (ME), and ratio of mixing index to pressure drop (ME/ΔP) calculated.

Device	Mixing Time (MT) [s]	Mean Residence Time (MRT) [s]	Trajectory Length (TL) [mm]	Pressure Drop (ΔP) [Pa]	Mixing Efficiency (ME)	ME/ΔP [Pa^−1^]
Chambers	12.76 ± 1.13	2.2323 ± 0.0004	90.7675 ± 0.0002	0.258 ± 0.003	87.60 ± 0.53%	2.94 ± 0.08
SARS	4.61 ± 0.70	2.7123 ± 0.0007	122.0137 ± 0.0678	0.577 ± 0.043	79.70 ± 0.94%	1.52 ± 0.16
Serpentine	7.95 ± 0.92	4.4304 ± 0.0001	216.6703 ± 0.0076	0.465 ± 0.030	77.96 ± 1.49%	1.71 ± 0.06
Circular Features	3.85 ± 0.15	1.8139 ± 0.0002	127.2851 ± 0.0002	0.627 ± 0.031	75.86 ± 0.12%	1.24 ± 0.03

## Supplementary Materials

The following are available online at https://www.mdpi.com/article/10.3390/mi12111377/s1, Table S1: Summary of parameters for the Multiphysics simulations to produce GUVs, Figure S1: Mesh convergence and sensitivity analysis of double emulsion generation Multiphysics simulation, Table S2: Summary of parameters for the Multiphysics simulations of encapsulation, Figure S2: Mesh convergence analysis of the four micromixers for the Multiphysics simulations of encapsulation, Figure S3: Volume fraction of the dispersed phase and velocity field for each FRR in the micromixers Multiphysics simulations of encapsulation, Figure S4: In silico mixing behavior along mixer length, Figure S5: Sensitivity analysis for the FRR and TFR of the micromixers Multiphysics simulations of encapsulation, Table S3: Summary of fluidic parameters for the experimental characterization of GUVs synthesis, Figure S6: SLS measurements of the GUVs synthesized at different FRR and LO phase concentrations, Figure S7: Visualization of the colorimetric mixing efficiency calculation, Figure S8: Comparison of in silico and in vitro mixing behavior for the micromixers, Video S1: Double emulsion formation at 1:10:30 FRR, Video S2: Double emulsion formation at 1:20:60 FRR, Video S3: Double emulsion formation at 1:30:90 FRR, Script S1: Supplementary python script for colorimetric mixing efficiency calculation.
