# Supplementary material for: Design and Manufacture of a Low-Cost Microfluidic System for the Synthesis of Giant Liposomes for the Encapsulation of Yeast Homologues: Applications in the Screening of Membrane-Active Peptide Libraries
Source: Micromachines (Basel). 2021 Nov 10;12(11):1377. doi: 10.3390/mi12111377 (PMC8619280; doi:10.3390/mi12111377)
Supplement: Supplementary file 1 [file micromachines-12-01377-s001.zip › micromachines-1437355-supplementary.pdf]

micromachines

Supplementary Materials

Design and Manufacture of a Low-Cost Microfluidic System for the Synthesis of Giant Liposomes for the Encapsulation of Yeast Homologues: Applications in the Screening of Membrane-Active Peptide Libraries

Saúl C. Gómez [1,†], Valentina Quezada [1,†], Isabella Quiroz [1], Carolina Muñoz-Camargo [1], Johann F. Osma [2,*], Luis H. Reyes [3,*] and Juan C. Cruz [1,*]

1 Department of Biomedical Engineering, Universidad de los Andes, Cra. 1E No. 19a-40, Bogotá 111711, Colombia; sc.gomez11@uniandes.edu.co (S.C.G.); v.quezada@uniandes.edu.co (V.Q.); i.quiroz@uniandes.edu.co (I.Q.); c.munoz2016@uniandes.edu.co (C.M.-C.)
2 Department of Electrical and Electronic Engineering, Universidad de los Andes, Cra. 1E No. 19a-40, Bogotá 111711, Colombia
3 Department of Food and Chemical Engineering, Universidad de los Andes, Cra. 1E No. 19a-40, Bogotá 111711, Colombia
* Correspondence: jf.osma43@uniandes.edu.co (J.F.O.); lh.reyes@uniandes.edu.co (L.H.R.); jc.cruz@uniandes.edu.co (J.C.C.); Tel.: +57-1-3394949 (ext. 1789) (J.C.C.)
† Co-first author, these authors contributed equally to this work.

Citation: Gómez, S.C.; Quezada, V.; Quiroz, I.; Muñoz-Camargo, C.; Osma, J.F.; Reyes, L.H.; Cruz, J.C. Design and Manufacture of a Low-Cost Microfluidic System for the Synthesis of Giant Liposomes for the Encapsulation of Yeast Homologues: Applications in the Screening of Membrane-Active Peptide Libraries. *Micromachines* **2021**, *12*, 1377. https://doi.org/10.3390/mi12111377

Academic Editor: Palaniappan Sethu

Received: 11 October 2021
Accepted: 6 November 2021
Published: 8 November 2021

Publisher's Note: MDPI stays neutral with regard to jurisdictional claims in published maps and institutional affiliations.

Copyright: © 2021 by the authors. Licensee MDPI, Basel, Switzerland. This article is an open access article distributed under the terms and conditions of the Creative Commons Attribution (CC BY) license (http://creativecommons.org/licenses/by/4.0/).

SI1. Double Emulsion Generation Simulation. Two-phase Flow, Level-set model in COMSOL Multiphysics®

Table S1. Summary of parameters for the Multiphysics simulations to produce GUVs.

Parameter	Value	Units
V_{IA}	0.001851853	m/s
V_{LO}	0.018518533	m/s
V_{OA}	0.0555556	m/s
FRR	1:10:30	
TFR	0.075926	m/s

(a) **(b)**

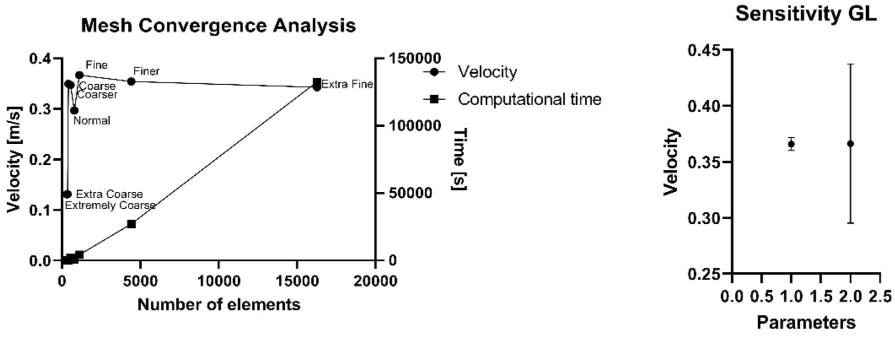

Figure S1. (a) Mesh convergence analysis for the velocity field and the computational time and (b) sensitivity analisys for FRR and TFR.

SI2. Yeast Encapsulation by Direct Interaction Simulation. Mixture Model in COMSOL Multiphysics®

Table S2. Summary of parameters for the Multiphysics simulations of encapsulation.

Parameter	Value	Units
FRR	1:1 – 2:1 – 3:1	-
TFR	0.084	m/s
Particle diameter (d_p)	50	µm
Maximum packaging concentration (ϕ_{max})	0.2	-
Minimum volume fraction – Dispersed phase (ϕ_d)	0.2	-

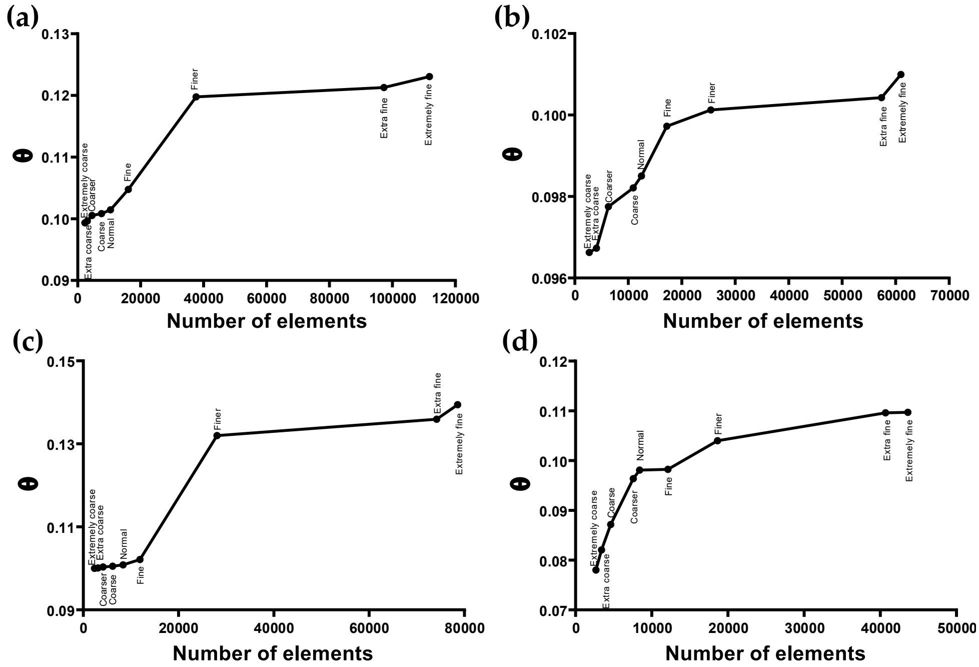

Figure S2. Mesh convergence analysis for the volume fraction dispersed phase (θ) of the micromixers: (a) Chambers; (b) Circular features; (c) SARS; (d) Serpentine.

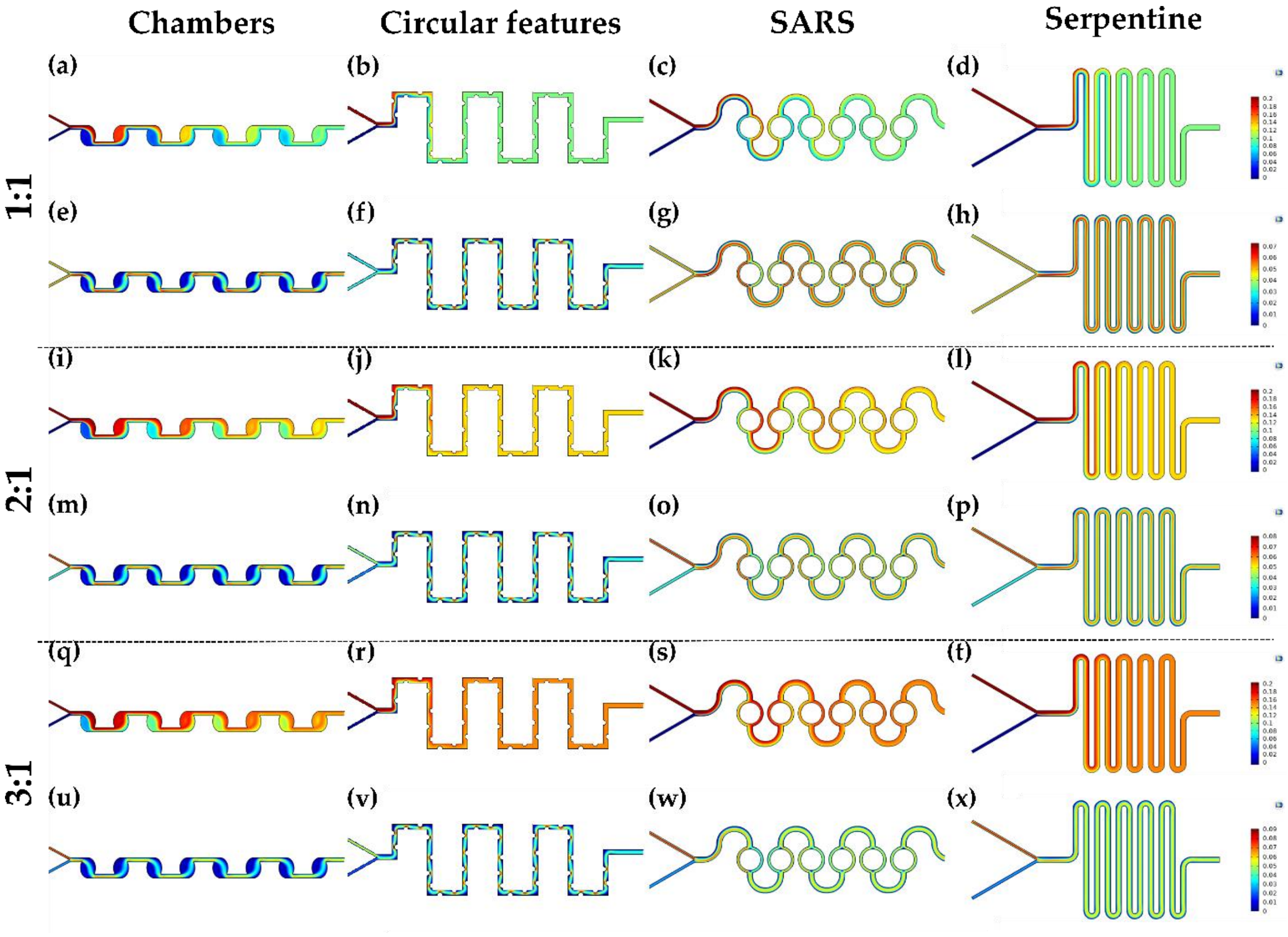

Figure S3. *In silico* results for the volume fraction of the dispersed phase (θ) (**a-d; i-l; q-t**) and the velocity field (**e-h; m-p; u-x**) (VF) for each FRR in the micromixers.

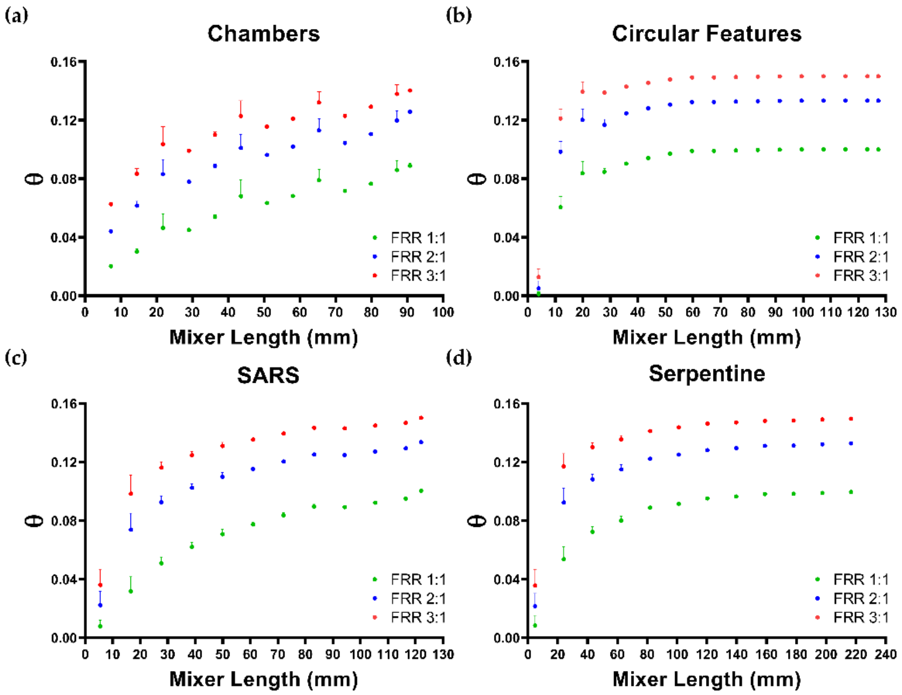

Figure S4. *In silico* mixing behavior: (**a**) Chambers; (**b**) Circular features; (**c**) SARS; (**d**) Serpentine.

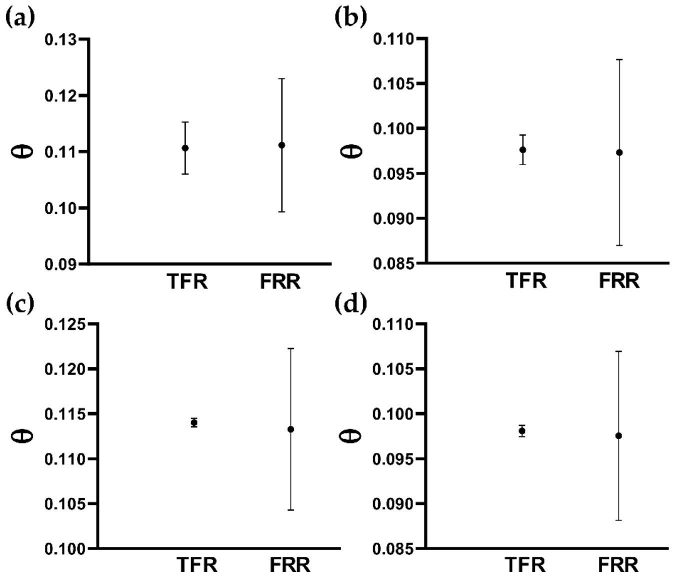

Figure S5. Sensitivity analysis for the FRR and TFR micromixers parameters: (**a**) Chambers; (**b**) Circular features; (**c**) SARS; (**d**) Serpentine.

SI3. Low-cost Octanol-assisted Liposomes Assembly FRR Characterization

Table S3. Summary of fluidic parameters for the experimental characterization of GUVs synthesis.

FRR	Flow rate [mL/h]	TFR [mL/h]
1:10:30	Q_{IA}: 10; Q_{LO}: 100; Q_{OA}: 300	410
1:20:60	Q_{IA}: 10; Q_{LO}: 200; Q_{OA}: 600	810
1:30:90	Q_{IA}: 6.7; Q_{LO}: 200.8; Q_{OA}: 602.5	810

SI4. Static light scattering (SLS) measurements of GUVs samples synthesized by the microfluidic system proposed

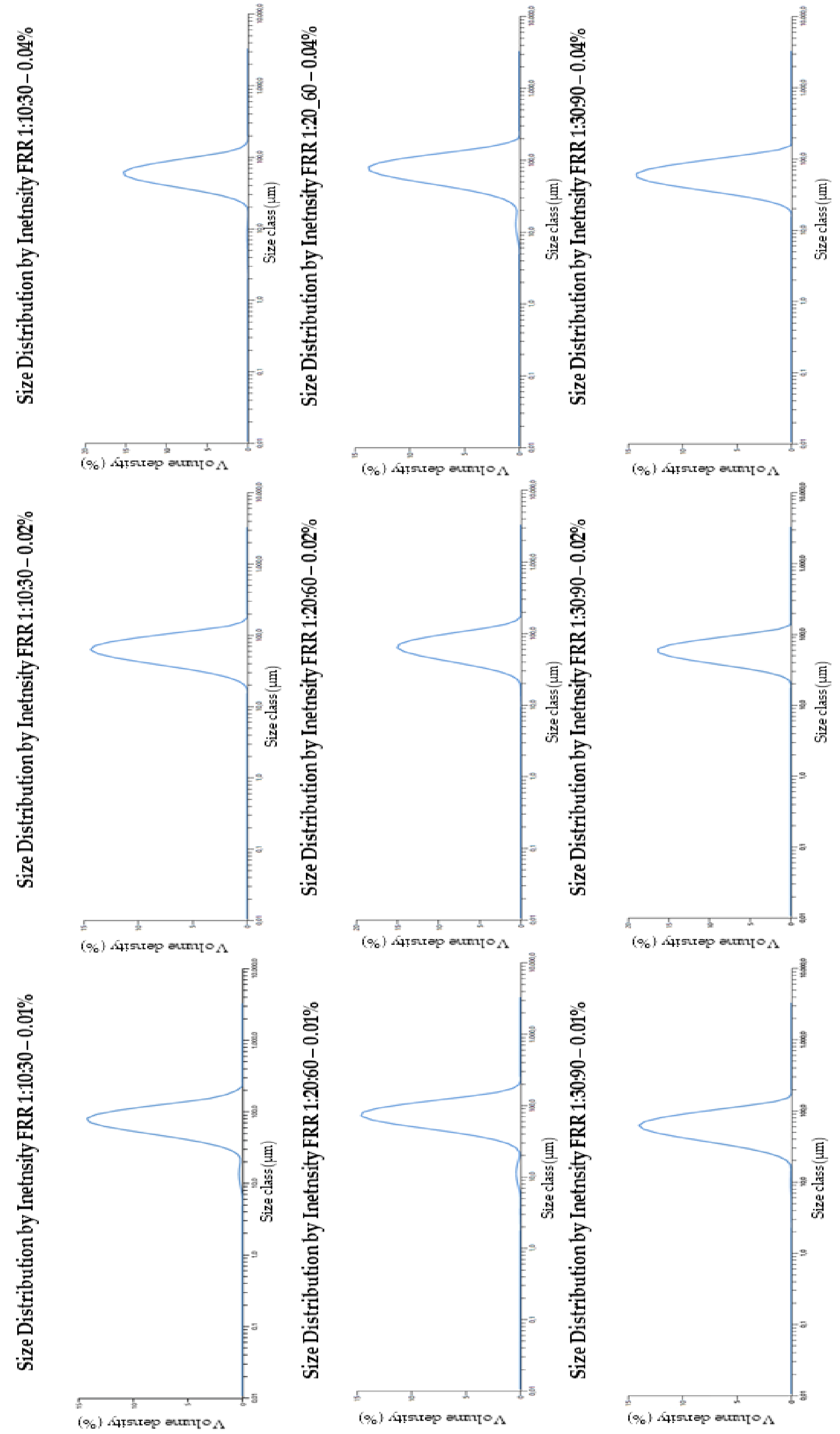
Size Distribution by Inetnsity FRR 1:10:30 – 0.04%
Size Distribution by Inetnsity FRR 1:20_60 – 0.04%
Size Distribution by Inetnsity FRR 1:30:90 – 0.04%
Size Distribution by Inetnsity FRR 1:10:30 – 0.02%
Size Distribution by Inetnsity FRR 1:20:60 – 0.02%
Size Distribution by Inetnsity FRR 1:30:90 – 0.02%
Size Distribution by Inetnsity FRR 1:10:30 – 0.01%
Size Distribution by Inetnsity FRR 1:20:60 – 0.01%
Size Distribution by Inetnsity FRR 1:30:90 – 0.01%
Size class (μm)
Volume density (%)

Figure S6. SLS measurements of GUVs samples synthesized varying the flow rate ratio (FRR) and the concentration of the LO phase.

SI5. Colorimetric Mixing Efficiency Test vs. *In silico* approach

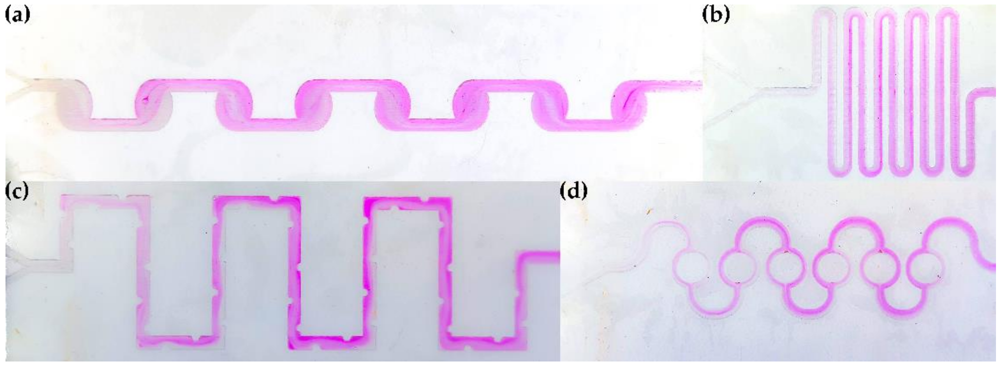

Figure S7. Image acquisition for the mixture efficiency calculation.

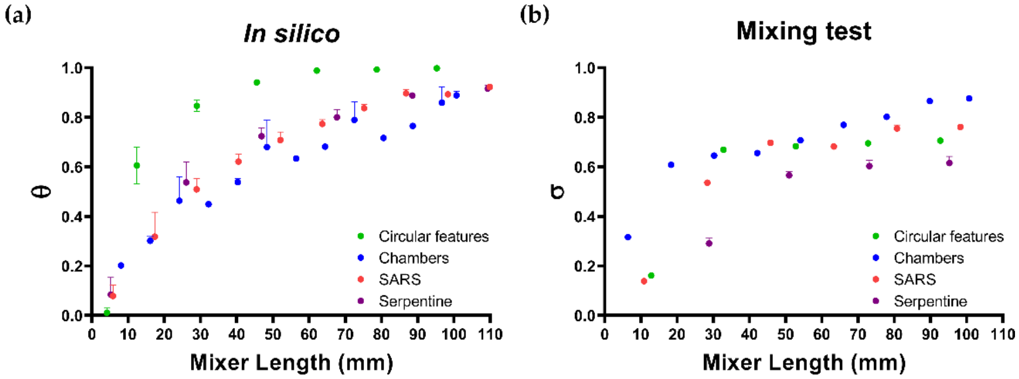

Figure S8. Comparison of *in silico* mixing behavior for FRR 1:1 and experimentally for the micromixers.
